# Lenvatinib, an angiogenesis inhibitor targeting VEGFR/FGFR, shows broad antitumor activity in human tumor xenograft models associated with microvessel density and pericyte coverage

**DOI:** 10.1186/2045-824X-6-18

**Published:** 2014-09-06

**Authors:** Yuji Yamamoto, Junji Matsui, Tomohiro Matsushima, Hiroshi Obaishi, Kazuki Miyazaki, Katsuji Nakamura, Osamu Tohyama, Taro Semba, Atsumi Yamaguchi, Sachi Suzuki Hoshi, Fusayo Mimura, Toru Haneda, Yoshio Fukuda, Jun-ichi Kamata, Keiko Takahashi, Masayuki Matsukura, Toshiaki Wakabayashi, Makoto Asada, Ken-ichi Nomoto, Tatsuo Watanabe, Zoltan Dezso, Kentaro Yoshimatsu, Yasuhiro Funahashi, Akihiko Tsuruoka

**Affiliations:** 1 Oncology Product Creation Unit, Eisai Product Creation Systems, Eisai Co., Ltd., 5-1-3 Tokodai, Tsukuba, Ibaraki 300-2635, Japan; 2 Biomarkers and Personalized Medicine Core Function Unit, Eisai Product Creation Systems, Eisai Co., Ltd., 5-1-3 Tokodai, Tsukuba, Ibaraki 300-2635, Japan; 3 Biomarkers and Personalized Medicine Core Function Unit, Eisai Product Creation Systems, Eisai Inc., 4 Corporate Drive, Andover, MA 01810, U.S.A

**Keywords:** Lenvatinib, VEGFR2 kinase inhibitor, FGFR kinase inhibitor, Pericyte coverage, Microvessel density

## Abstract

**Background:**

Lenvatinib is an oral inhibitor of multiple receptor tyrosine kinases (RTKs) targeting vascular endothelial growth factor receptor (VEGFR1-3), fibroblast growth factor receptor (FGFR1-4), platelet growth factor receptor α (PDGFR α), RET and KIT. Antiangiogenesis activity of lenvatinib in VEGF- and FGF-driven angiogenesis models in both in vitro and in vivo was determined. Roles of tumor vasculature (microvessel density (MVD) and pericyte coverage) as biomarkers for lenvatinib were also examined in this study.

**Method:**

We evaluated antiangiogenesis activity of lenvatinib against VEGF- and FGF-driven proliferation and tube formation of HUVECs in vitro. Effects of lenvatinib on in vivo angiogenesis, which was enhanced by overexpressed VEGF or FGF in human pancreatic cancer KP-1 cells, were examined in the mouse dorsal air sac assay. We determined antitumor activity of lenvatinib in a broad panel of human tumor xenograft models to test if vascular score, which consisted of high MVD and low pericyte coverage, was associated with sensitivity to lenvatinib treatment. Vascular score was also analyzed using human tumor specimens with 18 different types of human primary tumors.

**Result:**

Lenvatinib inhibited VEGF- and FGF-driven proliferation and tube formation of HUVECs in vitro. In vivo angiogenesis induced by overexpressed VEGF (KP-1/VEGF transfectants) or FGF (KP-1/FGF transfectants) was significantly suppressed with oral treatments of lenvatinib. Lenvatinib showed significant antitumor activity in KP-1/VEGF and five 5 of 7 different types of human tumor xenograft models at between 1 to 100 mg/kg. We divided 19 human tumor xenograft models into lenvatinib-sensitive (tumor-shrinkage) and relatively resistant (slow-growth) subgroups based on sensitivity to lenvatinib treatments at 100 mg/kg. IHC analysis showed that vascular score was significantly higher in sensitive subgroup than relatively resistant subgroup (p < 0.0004). Among 18 types of human primary tumors, kidney cancer had the highest MVD, while liver cancer had the lowest pericyte coverage, and cancers in Kidney and Stomach had highest vascular score.

**Conclusion:**

These results indicated that Lenvatinib inhibited VEGF- and FGF-driven angiogenesis and showed a broad spectrum of antitumor activity with a wide therapeutic window. MVD and pericyte-coverage of tumor vasculature might be biomarkers and suggest cases that would respond for lenvatinib therapy.

## Background

Angiogenesis, the formation of new blood vessels, has physiologic and pathologic roles [1]. RTK signaling pathways have been identified as crucial regulators of angiogenesis, including the signaling pathways of VEGFR, FGFR, HGFR, PDGFR, TIE2 and EPH expressed in endothelial or vascular mural cells [1]. The VEGF-signaling pathway is the key regulator of tumor growth and metastasis and consists of five ligands (VEGFA-D and placental growth factor (PlGF)) and three RTKs (VEGFR1, VEGFR2, VEGFR3) [2]. Bevacizumab (a monoclonal antibody against VEGFA) was the first angiogenesis inhibitor, and was approved for the treatment of patients with metastatic colon cancer based on its survival benefit [3]. It is also used to treat non-small cell lung cancer (NSCLC), renal cell carcinoma (RCC), glioblastoma multiforme (GBM) and breast cancer [4]. The use of bevacizumab confirmed that blocking the VEGF-signaling pathway is a feasible approach to cancer therapy.

VEGFR2 induces major phenotypic changes of endothelial cells in angiogenesis, including proliferation, migration, survival, and tube formation [5-7]. Several small-molecule inhibitors of VEGFR2 kinase have been approved for treatments of multiple cancer types, such as sunitinib (RCC, gastrointestinal stromal tumor (GIST)) [8,9], sorafenib (RCC, HCC, differentiated thyroid cancer (DTC)) [10-12], pazopanib (RCC, soft-tissue sarcoma (STS)) [13,14], axitinib (RCC) [15], vandetanib (medullary thyroid cancer (MTC)) [16] and regorafnib (colorectal cancer (CRC), GIST) [17,18]. The approved indications for bevacizumab and VEGFR2 tyrosine kinase inhibitor (TKI) are similar for CRC and RCC, but different for breast cancer, HCC, NSCLC, GBM, DTC, MTC and STS. Among the five ligands of the VEGF-signaling pathway (VEGFA-D and PIGF), bevacizumab targets only VEGFA. VEGFR1 predominantly mediates chemotactic activity in monocytes and macrophages [19], and mobilization of bone-marrow-derived endothelial and hematopoietic stem cells [20]. VEGFR3 is expressed by the lymphatic endothelium and promotes tumor lymphangiogenesis and tumor spread through lymphatic vessels [21]. Thus, the inhibition of signal transduction via multiple VEGFRs may be a promising therapeutic strategy. The development of novel inhibitors of multi-targeted RTKs in addition to VEGFR2 is still required to improve cancer therapy in the clinic [22], since one of the resistance mechanisms involved is the up-regulation of alternative pro-angiogenic signaling pathways that include FGF/FGFR, angiopoietin/TIE2 and ephrin/EPH [23].

Antiangiogenesis therapy with antibody against VEGF or inhibitors of multiple RTKs targeting VEGFR2 improves the survival of patients with a variety of advanced cancers. However, the durations of treatment are limited due to acquired resistance, and sub-groups of patients do not respond due to intrinsic resistance [23]. Surrogate biomarkers of those angiogenesis inhibitors might help improve the selection of appropriate patients and contribute to decisions regarding whether to continue antiangiogenesis therapy. The biomarkers for identifying responsive patients for antiangiogenesis therapy included plasma proteins [24], circulating endothelial cells [25,26], and novel imaging techniques [27], but no reliable predictive biomarkers have been established. Interactions between endothelial cells and vascular mural cells (e.g., pericytes) have been studied and in light of the finding that bevacitzumab increased the numbers of pericyte-covered vessels after one-shot treatment for human colorectal cancer [28,29], it is likely that the extent of the interaction of tumor endothelial cells with pericytes is relevant to the responsiveness to antiangiogenesis therapy. However it has not been proven yet that those interactions predict antitumor activity of antiangiogenesis therapy. Examinations of these interactions would thus be important in the development of predictive biomarkers for antiangiogenesis therapy in both preclinical and clinical cancer.

We previously reported a novel multi-targeted VEGFR2 TKI, lenvatinib (E7080), which inhibited KIT-dependent angiogenesis [26] and VEGFR3-related lymphangiogenesis [30]. Lenvatinib showed the activity against multiple types of cancer in a phase I study [25,31] and phase II/III clinical studies in patients with such cancers as DTC, MTC, HCC, melanoma, and endometrial cancer are currently in progress. In this study, we investigated the pharmacologic profile of lenvatinib and we determine the antiangiogenesis activity in VEGF- and FGF-driven angiogenesis assays. Next, antitumor activity of lenvatinib was explored in a panel of various human tumor xenograft models in order to identify biomarkers for predicting the response to lenvatinib. MVD and pericyte coverage were determined by immunohistochemical (IHC) analysis to perform vascular score analysis as candidate biomarkers and an association of vascular score with the antitumor activity of lenvatinib was analyzed. In addition, mRNA expression levels of genes related to angiogenesis by qPCR were correlated with antitumor activity of lenvatinib. Lastly vascular score analysis was also performed using human tumor tissue specimens, if vascular score is able to identify tumor vascular phenotypes of human cancer.

## Materials and methods

### Chemical synthesis of lenvatinib

**Figure 1:**
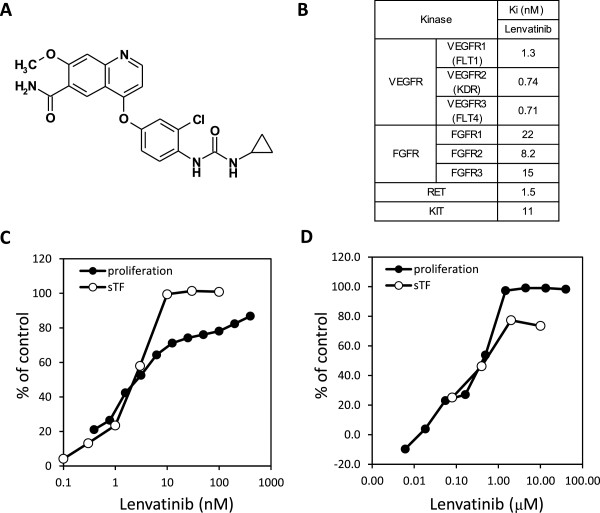
**Kinase inhibitory activity and antiangiogenic activity of lenvatinib in vitro. A**: Chemical structure of lenvatinib. **B**: Ki values of lenvatinib. **C**: Effects of lenvatinib on the VEGF-induced proliferation and tube formation of HUVEC. **D**: Effects on the FGF-induced proliferation and tube formation of HUVEC. sTF assay; sandwich tube formation assay.

The chemical name of lenvatinib is [4-[3-Chloro-4-(N’-cyclopropylureido)phenoxy]-7-methoxyquinoline-6-carboxamide], and the structural formula is shown in Figure 1A. The compound was synthesized by Eisai Co., Ltd., and its chemical identity was established by nuclear magnetic resonance and mass spectroscopy.

### Cell lines

Tumor cell lines were purchased from the American Type Culture Collection (Rockville, MD), except for human pancreatic tumor KP-1 and FEM cells, which were from the National Kyusyu Cancer Center (Fukuoka, Japan) and Prof. Ostein Fodstad at Norwegian Radium Hospital Research Foundation, respectively. KP-1 transfectants were prepared by stable transfection (Effecten transfection reagent; Qiagen, Tokyo, Japan) of pcDNA3.1 vectors (Life technologies, Tokyo, Japan) coding either human VEGF121 (KP-1/VEGF) or mouse FGF4 (KP/FGF) into KP-1 cells and selected by G418 (Life technologies). VEGF121 lacks a heparin binding site and is therefore more diffusible than VEGF165. FGF4 has a signal peptide, but FGF1 and FGF2 do not. Therefore, we selected VEGF121 and FGF4 for overexpression in this study, since in vivo angiogenesis in Dorsal Air Sac (DAS) assay is driven by secreted angiogenic factors from tumor cells packed into the Millipore chambers. Tumor cell lines were maintained in RPMI1640 medium containing 10% heat-inactivated fetal bovine serum (FBS). Human umbilical vein endothelial cells (HUVECs) were isolated from a human umbilical cord by a method described previously [32] and cultured using an EGM-2 BulletKit (Sanko Junyaku, Tokyo, Japan).

### Animals

Balb/c nude mice (Charles River Laboratories, Yokohama, Japan) were used for human tumor xenograft models. Animal experiments were conducted in accordance with the Institutional Animal Care and Use Committee guidelines of Eisai Co., Ltd.

### In vitro kinase assay

Tyrosine kinase assays were performed by an ELISA and Off-chip Mobility Shift Assay (MSA) by Carna Biosciences, Inc. (Kobe, Japan).

### Proliferation assay

Tumor cells were plated at 1 to 2 × 10^3^ cells/well on 96-well plates in 0.1 ml of RPMI 1640 containing 10% FBS. After 24 h, lenvatinib or vehicle was added, and 2 or 3 days after drug treatments, The ratios of surviving cells were calculated by measuring the absorbance of 3-(4,5-dimethylthiazol-2-yl)-2,5-diphenyltetrazolium bromide (MTT) in each well and expressing it relative to the absorbance in the control wells. The HUVEC proliferation assay were performed by a method described previously [32]. Briefly, HUVECs were seeded on type I-collagen coated 96-well plates at 0.6 × 10^3^ cells with serum free medium (SFM) (Invitrogen, Carlsbad, CA) containing 2% FBS (2% FBS-SFM) and an aliquot of lenvatinib with VEGF (Genzyme, Cambridge, MA) or FGF2 (Sigma-Aldrich, St. Louis, MO) (20 ng/mL) in 2% FBS-SFM was added. After incubation for 3 days, cell viability was determined by measuring the absorbance of WST-1 reagent (DOJINDO, Kumamoto, Japan). Percent inhibition of cell growth was determined by the formula below: Inhibition (%) = Mean value of OD of control wells - OD of treated wells * 100/Mean value of OD of control wells. Each value is expressed as the mean. An IC_50_ value was determined from the curves representing growth inhibition activity versus lenvatinib concentration. All experiments were done in either duplicate or quadruplicate.

### In vitro angiogenesis assay

In vitro sandwich tube formation (sTF) assay, HUVECs in SFM containing EGF (10 ng/mL) with VEGF (20 ng/mL) or FGF2 (20 ng/ml) were plated onto the 1st layer of collagen gel (Nitta Gelatin, Tokyo, Japan) at 1.0 × 10^5^ cells in 24-well plates and a 2nd layer of collagen gel was added onto the HUVECs. An aliquot of lenvatinib in SFM containing EGF with VEGF- or FGF2 was added at the indicated dose. After 4 days, MTT solution was added and photomicrographs of tube formation were taken with a light microscope. The tube length of capillaries was measured using image analysis (Angiogenesis Image Analyzer, ver. 1.04; Kurabo, Osaka, Japan).

### Mouse dorsal air sac (DAS) assay

An in vivo angiogenesis assay in mice was performed as described previously [32] with minor changes. Millipore chambers (Millipore, Bedford, MA) were packed with 1.0 × 10^7^ KP-1 mock, KP-1/VEGF or KP-1/FGF transfectants and transplanted into the dorsal air sac of C57BL/6 mice (n = 3–5 each, with experiments performed in triplicate). For a pseudo-operation, collagen I gel was injected into the Millipore chambers. Photographs were taken 4 days after implantation.

### Subcutaneous (s.c.) xenograft models

Tumor cells were injected subcutaneously into the right flank region of 7-week-old nude mice. When the mean tumor volume reached approximately 100–300 mm^3^, tumor-bearing mice were randomized and the administration of either vehicle or lenvatinib was started (day 1). Tumor diameters were measured with a vernier caliper and body weight was measured twice weekly. The tumor volume (mm^3^) was calculated using the formula: length (mm) × width (mm)^2^ × 1/2. The antitumor activity of lenvatinib was calculated as a ΔT/C (% of control for Δgrowth) after the administration period. ΔT/C was calculated according to the formula: (ΔT/ΔC) × 100. ΔT and ΔC are the changes in tumor volume (Δgrowth) for the treated and vehicle/control groups, respectively. When the tumor volume was reduced, the ΔT/C values were calculated by the following formula: ΔT/C (%) = (TVn - TV1)/TV1 × 100, where TVn is the tumor volume of treated mice at day n. The number of mice is ten for control group in Table 1 and five for each group in other experiments. Mice in control group were treated with vehicle.

**Table 1:** Summary of antitumor activity of lenvatinib in seven subcutaneous xenograft models

**Tumor cell**	**IC**_**50**_^**1)**^**(μM)**	**TV**^**2)**^**(mm**^**3**^**)**	**ΔTV (%)**^**3)**^**lenvatinib (mg/kg)**				
			**1**	**3**	**10**	**30**	**100**
MDA-MB 435^4)^ (Melanoma)	24.2	180	55.7*	56.9*	40.2*	21.2*	25.7*
MIApaca-II^5)^ (Pancreas)	33.8	250	56.1*	49.9*	30.1*	15.3*	12.5*
H460^5)^ (Lung)	26.3	110	65.4	34.9*	16.3*	16.4*	-0.8*
SK-OV-3^5)^ (Ovary)	26.4	230	52.3*	39.6*	12.2*	8.3*	-15.6*
Colo205^5)^ (Colon)	27.5	320	43.8*	23.1*	12.8*	-4.5*	-21.3*
A431^5)^ (Epidermoid)	30.8	310	41.7	29.4*	0.0*	-7.5*	-32.9*
DU145^4)^ (Prostate)	30.8	160	-7.5*	-42.2*	-65.4*	-72.0*	-78.6*

^1)^In vitro anti-proliferative activity of lenvatinib.

^2)^Lenvatinib treatments started at indicated tumor volumes.

^3)^ΔT/C of individual mice was determined first, and then an mean value was calculated.

^4)^Lenvatinib was orally administered twice daily for 28 days.

^5)^Lenvatinib was orally administered twice daily for 14 days.

*p < 0.05 versus vehicle control.

### IHC analysis of MVD and pericyte coverage

We defined pericyte-covered vessels as endothelial cells were fully surrounded or partially surrounded by αSMA positive cells as described previously [33,34]. Briefly, 8-μm frozen sections were fixed with cold acetone and immunostained with rat anti-mouse CD31 monoclonal antibody (BD Biosciences, Franklin Lakes, NJ) and mouse anti-α-SMA monoclonal antibody (Sigma-Aldrich) by the ABC and LSAB methods using a Vectastain ABC kit (Funakoshi, Tokyo, Japan) and LSAB kit (Dako, Glostrup, Denmark). Adjacent sections were routinely stained with hematoxylin and eosin. All histological specimens were viewed under a CCD Hyper Scope (Keyence, Osaka, Japan) and analyzed using Image Tool software (Image Tool, Roswell, GA). MVD was determined as the mean of four or five fields per cross-section. Pericyte coverage was calculated based on the ratio of the double-staining vessels per total vessel count in the hotspot areas. Human tumor specimens were purchased from SuperBioChips (Seoul, South Korea). The human tumor specimens were double-stained with mouse anti-human CD31 monoclonal antibody (Dako) and alkaline phosphatase-labeled mouse anti-α-SMA monoclonal antibody (Sigma-Aldrich) using an Envision HRP kit (Dako) and the LSAB kit.

### TaqMan® Low-Density Array (TLDA) analysis of implanted tumors

Total RNA was isolated from xenografted tumors at a size of approximately 100–300 mm^3^ using an RNeasy kit (Qiagen, Valencia, CA). Reverse transcription was performed for 59 genes related to angiogenesis using Multiscribe™ Reverse Transcriptase (Invitrogen), and the quantitative real-time polymerase chain reaction (qPCR) analysis was performed using a TaqMan® Low-Density Array (Invitrogen) (Additional file 1).

### Statistical analysis

For the statistical analysis of the activity in the s.c. xenograft models and mouse DAS models, a repeated measures analysis of variance (ANOVA) was used, followed by a Dunnett-type multiple comparison test. Statistical analyses for MVD and % of pericyte coverage were performed by student *t*-test and vascular score analysis was performed by Wilcoxon test. Correlative analysis of mRNA with ΔT/C was performed by Spearman’s rank correlation test and association with sensitivity was done by Mann–Whitney *u* test. Results were considered significant at p < 0.05.

## Results

### Kinase inhibitory profile and effects of lenvatinib in in vitro VEGF- and FGF-driven proliferation and tube formation of HUVEC

Lenvatinib is a novel quinoline derivative containing an aryl urea moiety (Figure 1A). The kinase inhibitory profile of lenvatinib was determined by biochemical kinase assays for tyrosine kinases (Figure 1B). Lenvatinib strongly inhibited VEGFR1, 2, 3 RTK (Ki = 1.3, 0.74, 0.71 nM, respectively), and also exhibited inhibitory activities against FGFR1, 2, 3 RTK (Ki = 22, 8.2, 15 nM, respectively) in addition to RET and KIT (Ki = 1.5 and 11nM, respectively). We previously reported that lenvatinib inhibited angiogenesis through activated KIT [26]. Since lenvatinib showed an equivalent inhibition against FGFR RTK to KIT, we determined effects of lenvatinib on HUVECs stimulated with FGF-2 besides VEGF. Lenvatinib inhibited VEGF- induced proliferation and tube formation of HUVECs with IC_50_ values of 3.4 and 2.7 nM, respectively (Figure 1C). Lenvatinib also inhibited FGF-2 induced proliferation and tube formation of HUVECs with IC_50_ values of 410 and 590 nM, respectively (Figure 1D).

### Antiangiogenesis and antitumor activity of lenvatinib in VEGF- and FGF-dependent angiogenesis models

**Figure 2:**
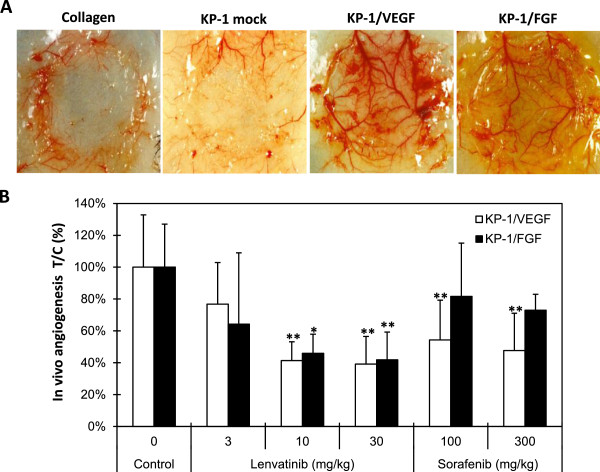
**Effects of lenvatinib on in vivo angiogenesis induced by KP-1/VEGF and KP-1/FGF transfectants. (A)** In vivo angiogenesis in mouse DAS assay. Angiogenesis was induced by overexpressed human VEGF121 (KP-1/VEGF) or mouse FGF4 (KP-1/FGF) in human pancreatic cancer KP-1 cells at the mouse dorsal skin. Representative photographs are shown. **(B)** Effect of lenvatinib and sorafenib on the VEGF- and FGF-driven in vivo angiogenesis in mouse DAS assay. Compounds were administered orally once daily for 4 days at the indicated doses. Data are the mean ± std. *: p < 0.05 and **: p < 0.01 compared to vehicle.

We established tumor models in which the in vivo tumor growth was promoted by VEGF- and FGF-induced angiogenesis to evaluate both antiangiogenesis and antitumor activity of lenvatinib in mice. Human pancreatic cancer KP-1 cells exhibited a minimum angiogenic activity in the mouse DAS assay (Figure 2A and Additional file 2A). KP-1 cells were stably transfected to overexpress either human VEGF121 (KP-1/VEGF) or mouse FGF-4 (KP-1/FGF) (Additional file 2B and Additional file 3A). There was no difference of in vitro growth rate between these transfectants (data not shown). Conditioned medium from KP-1/VEGF and KP-1/FGF cultures induced tube formation of HUVECs in the sTF assay (Additional file 2C). Consistent with the sTF assay, in vivo angiogenesis was induced by KP-1/VEGF and KP-1/FGF compared to KP-1 mock (Figure 2A and Additional file 2A). Lenvatinib at doses of 10 and 30 mg/kg significantly inhibited in vivo angiogenesis in both the KP-1/VEGF and KP-1/FGF models (Figure 2B), while sorafenib showed selective antiangiogenic activity in the KP-1/VEGF model at 100 and 300 mg/kg. This data suggested that lenvatinib inhibited both VEGF- and FGF-induced angiogenesis in mice.

**Figure 3:**
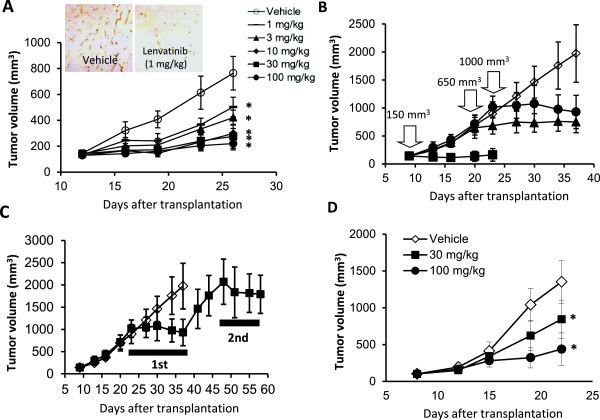
**Antitumor activity of lenvatinib against the KP-1/VEGF and KP-1/FGF transfectants in nude mice.** Lenvatinib was administered orally twice daily, when tumor volumes reached approximately 200 mm^3^**(A,C,D)**. Each group consisted of 5 mice. Data are the mean ± std. *p < 0.05 compared to vehicle. **(A-C)** the KP-1/VEGF xenograft model. **(D)** the KP-1/FGF xenograft model. **(A)** Antitumor activity of lenvatinib against KP-1/VEGF xenografts. Lenvatinib was administered at 1–100 mg/day for 14 days. Tumor tissues were resected on day 26 for IHC analysis. Tumor vessels were stained with anti-mouse CD31 antibody. Photographs were taken using a light microscope (x25) and representative images are shown. **(B)** Antitumor activity of lenvatinib in the advanced KP-1/VEGF xenograft model. Lenvatinib was administered at 100 mg/kg for either 14, 18 or 14 days, when the tumor size reached 150, 650 and 1000 mm^3^, respectively. **(C)** Antitumor activity of lenvatinib with an interval of treatments. Lenvatinib was administered at 100 mg/kg for 14 days in the 1st cycle and again given for 10 days in a 2nd cycle with 11 days interval between the 1st and 2nd cycles. (**D)** Antitumor activity of lenvatinib in the KP-1/FGF xenograft model. Lenvatinib was administered at 30 and 100 mg/kg for 14 days.

The in vivo tumor growth of KP-1/VEGF and KP-1/FGF transfectants was faster than that of KP-1 mock in nude mice (Additional file 3B), accompanied with selective overexpression of VEGF and FGF with increased angiogenesis (Additional file 3A). Lenvatinib did not potently inhibit the in vitro growth of KP-1/VEGF transfectants (IC_50_: 19.6 μM). In the KP-1/VEGF xenograft model, lenvatinib treatments at doses between 1 to 100 mg/kg resulted in significant inhibitions of tumor growth (Figure 3A). IHC analysis (CD31 staining) demonstrated lenvatinib inhibited angiogenesis at 1 mg/kg (Figure 3A) and resulted in 80% to 86% reduction of MVD compared to the control group at doses of 1 to 100 mg/kg (Additional file 4A). At a dose of 3 mg/kg, the inhibition of VEGFR2 phosphorylation lasted for 6 hrs and at a dose of 30 mg/kg, it was remarkably inhibited until 24 hrs after lenvatinib treatment (Additional file 4B). Lenvatinib inhibited VEGF-induced tyrosine phosphorylation of VEGFR2 in HUVECs with an IC_50_ value of 0.25 nM and also inhibited downstream signals, such as the phosphorylation of extracellular signal-regulated kinase (Erk)1/2 and Akt (Additional file 4C). These results indicated that lenvatinib inhibited VEGFR2 phosphorylation on tumor vessels in the KP-1/VEGF model. Next, we examined the antitumor activity of lenvatinib in advanced KP-1/VEGF transfectant models, in which large tumors were treated with lenvatinib (Figure 3B). Lenvatinib significantly inhibited tumor growth of KP-1/VEGF transfectants and had similar anti-tumor activity against large and small tumors. In addition, the second cycle of lenvatinib treatments resulted in a level of inhibitory activity similar to that seen in the first cycle (Figure 3C), suggesting that KP-1/VEGF xenografts did not acquire resistance to lenvatinib treatment. In the KP-1/FGF xenograft models, lenvatinib at doses of 30 and 100 mg/kg resulted in a dose-dependent inhibition of tumor growth (Figure 3D).

### Antitumor activity of lenvatinib in various types of human tumor xenograft models

Antitumor activity of lenvatinib was investigated using seven human tumor xenograft models of melanoma (MDA-MB 435), pancreatic (MIApaca-II), lung (H460), ovarian (SK-OV-3), colorectal (Colo205), epidermoid (A431) and prostate (DU145) cancers in nude mice (Table 1). Treatment with lenvatinib at doses of 1 to 100 mg/kg resulted in a dose-dependent inhibition of tumor growth in all seven tumor xenograft models and significantly inhibited tumor growth in 5 of 7 models even at 1 mg/kg. Lenvatinib at 100 mg/kg (maximum tolerated dose (MTD)) caused tumor shrinkage of more than 10% in 4 of 7 tumor xenograft models. Lenvatinib did not show potent inhibitory activity against the in vitro growth of these tumors (Table 1). Nude mice tolerated lenvatinib treatments well and only slight losses of body weights (10%<) at a dose of 100 mg/kg twice daily for 4 weeks was observed in 3 out of 7 models. These results indicated that lenvatinib had a wide therapeutic window in VEGF-dependent tumor models and caused tumor shrinkage at MTD dose in more than a half of various types of human tumor xenograft models.

### IHC analysis of tumor vasculature (MVD and pericyte coverage) as biomarkers associated with the antitumor activity of lenvatinib

**Figure 4:**
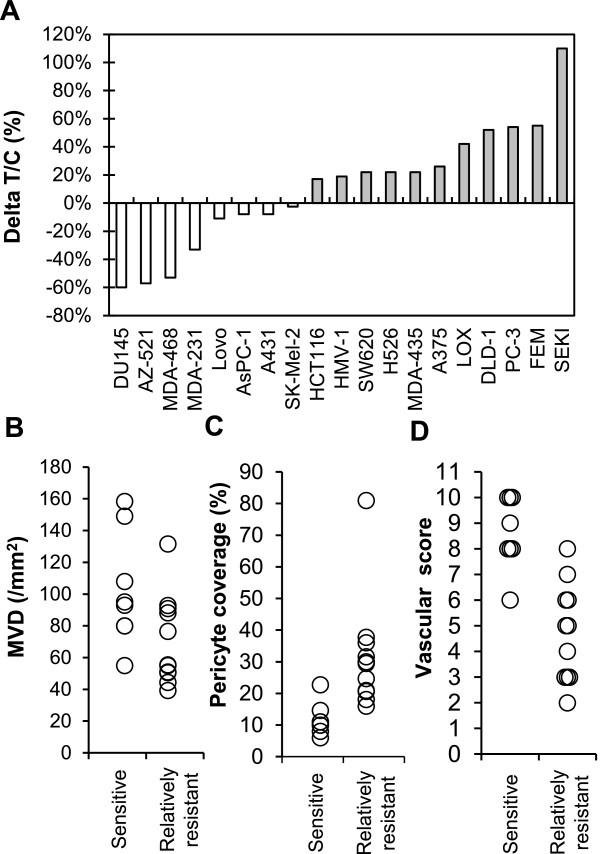
**Association of antitumor activity of lenvatinib with tumor vasculature in 19 human tumor xenograft models.** Lenvatinib was administered orally twice daily for 7 days, when tumor volumes reached approximately 100–300 mm^3^. Each group consisted of 5 mice. **(A)** Antitumor activity of lenvatinib in 19 human tumor xenograft models. The ΔT/C (%) was presented as a mean. □: The lenvatinib-sensitive group; ■: the lenvatinib-relatively resistant group. The relationship between the antitumor activity and MVD is shown in **(B)**, and that between the antitumor activity and the % of pericyte coverage of vessels is shown in **(C)**. Each symbol (○) indicates the mean of MVD or pericyte coverage in each tumor xenograft model. **(D)** Vascular score in the lenvatinib-sensitive and –relatively resistant groups. The vascular score was the sum of the MVD and pericyte coverage scores.

There are accumulating evidences that tumor vasculature evaluated by MVD and % of pericyte coverage has an impact on disease prognosis and/or acquired resistance to antiangiogenesis therapy in cancer patients [4,23]. Antitumor activity of lenvatinib was determined in an extended panel of 19 human tumor xenograft models (Figure 4A and Additional file 5) to explore tumor vasculature (MVD and % of pericyte coverage) as biomarkers to predict response to lenvatinib. Lenvatinib treatment was done at a dose of 100 mg/kg, since we detected tumor shrinkage in more than a half of 7 xenograft models at this dose (Table 1). Lenvatinib treatment resulted in tumor shrinkage in 8 tumor xenograft models, which were designated the “sensitive” tumors (ΔT/C < 0), while lenvatinib treatment caused slowing of tumor growth in 10 tumor xenograft models and had no effect in the SEKI model. These 11 tumors were designated as the “relatively resistant” tumors (ΔT/C > 0). We next analyzed MVD and % of pericyte coverage at pre-treatment (baseline) by IHC assay (CD31 for MVD and αSMA for pericyte coverage) (Additional file 5). There were significant associations of high MVD (p = 0.043) and low % of pericyte coverage (p = 0.0078) with antitumor activity of lenvatinib in the sensitive tumors compared to the relatively resistant tumors (Figure 4B and 4C). To further investigate the relationship of antitumor activity of lenvatinib to tumor vasculature, we performed an analysis using vascular score (Figure 4D) based on the sum of MVD score (ranging from 1 (low MVD) to 6 (high MVD)) and pericyte coverage score (ranging from 0 (high pericyte coverage) to 6 (low pericyte coverage)) (Additional file 6). There was more significant difference between the sensitive and relatively resistant tumors (p = 0.0004) with vascular score. These data suggested that tumor vasculature (MVD and % of pericyte coverage) was the biomarker of the response to lenvatinib and vascular score predicted antitumor activity of lenvatinib in preclinical 19 xenograft models.

### IHC analysis of MVD and % of pericyte coverage using human tumor specimens

**Figure 5:**
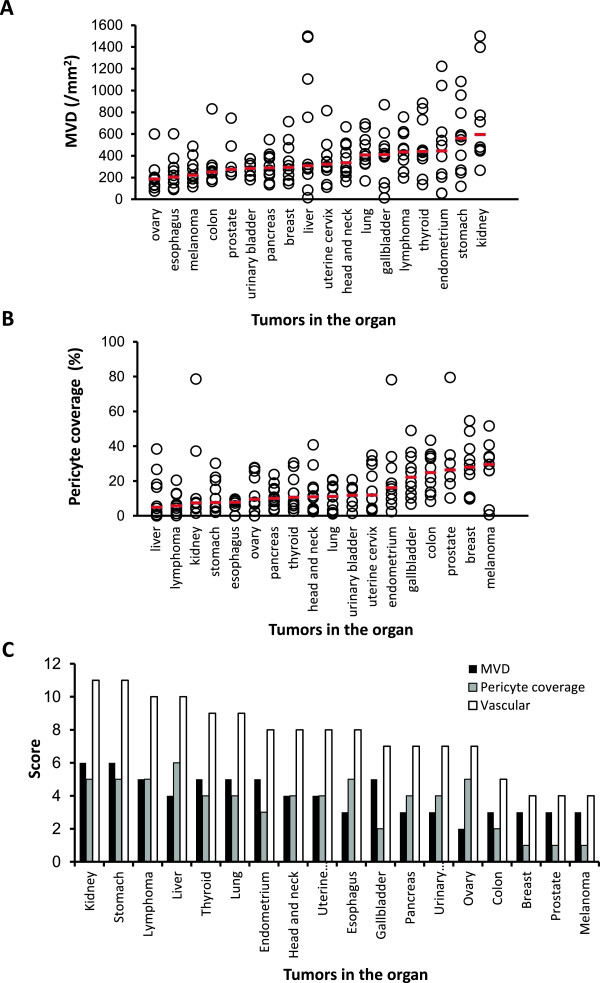
**IHC analysis of tumor vasculature in 18 different types of human tumor specimens.** Microvessel density (MVD), pericyte coverage and vascular scores were determined with IHC analysis by staining CD31 and αSMA among 18 types of human tumor specimens. Analysis was performed as described in materials and methods. Bars (red) indicated median values for MVD or % of pericyte coverage of each type of tumors. **(A)** MVD. **(B)** % of pericyte coverage. **(C)** Summary of MVD, pericyte coverage and vascular score.

We examined MVD and % of pericyte-coverage using human tumor tissues specimens (tissue micro-arrays) to determine if vascular score was able to define tumor vascular phenotypes in human cancer, as we did in human tumor xenograft models. We analyzed 18 different types of human primary tumor specimens (Additional file 7) by IHC analysis (CD31 for MVD and αSMA for % of pericyte coverate) (Figure 5A and 5B). The median MVD ranged from 185.5 to 595.5 per mm^2^ in all types of human tumor specimens and tumors in the kidney had the highest MVD (595.5 per mm^2^), whereas the median pericyte coverage ranged from 4.9% to 29.6% in all types of human tumor specimens and tumors in the liver had the lowest median pericyte coverage (4.9%) (Additional file 8). To analyze MVD combined with pericyte coverage, we performed vascular score analysis using the sum of MVD and pericyte coverage scores (Additional file 6B). Tumors in the kidney and stomach had the highest vascular scores at 11, followed by those in the liver and lymphoma at 10 and in the thyroid and lung at 9 (Figure 5C). These results suggested that IHC analyses of MVD and % of pericyte coverage with vascular score were considered as biomarkers for searching target types of cancers for lenvatinib and responses to lenvatinib treatments based on tumor vasculature.

### Preclinical correlative analysis of mRNA expression of genes related to angiogenesis with antitumor activity of lenvatinib

We performed qPCR analysis of gene expression related to angiogenesis within tumor tissues from a panel of 19 human tumor xenograft models, in which antitumor activity of lenvatinib was determined (Figure 4A). We associated mRNA expression levels with antitumor activity of lenvatinib in the sensitive and relatively-resistant sub-groups (Additional file 1A). Among the 59 genes examined, expression levels of high EGFR (p = 0.018, fold difference of median = 6.867) and low PlGF (p = 0.027, fold difference of median = 0.554) were significantly associated with antitumor activity in the sensitive sub-group. We also correlated mRNA expression levels with ΔT/C (Additional file 1B). High EGFR and KITLG expression levels (p = 0.014, 0.023 and r = 0.568, 0.533, respectively) and low PDGFRA and FGF7 expression levels (p = 0.010, 0.049 and r = -0.590, 0.470, respectively) were correlated with small ΔT/C.

## Discussion

In this study, we determined the inhibitory activity (Ki) of lenvatinib against VEGFR1-3, FGFR1-3, RET and KIT. Lenvatinib inhibited VEGF- and FGF2-driven proliferation and tube formation of HUVECs in vitro and also in vivo angiogenesis in mouse DAS assays using the KP-1/VEGF and KP-1/FGF, in which angiogenesis was induced by over-expressed angiogenic factors. Lenvatinib significantly inhibited the in vivo growth of KP-1/VEGF tumor, which was enhanced by VEGF-driven angiogenesis, accompanied by decreased MVD by IHC analysis. Lenvatinib showed significant antitumor activity from 1 mg/kg to 100 mg/kg in KP-1/VEGF models. This data suggested lenvatinib showed wide therapeutic windows in the highly VEGF-dependent model. Antitumor activity of lenvatinib was highly associated with vascular score (combined scores of high MVD and low pericyte coverage) in a panel of 19 human tumor xenograft models consisting of variety of cancer types.

Lenvatinib showed significant antiangiogenesis activity (decrease of MVD) in the KP-1/VEGF xenograft model at 1 mg/kg and at 10 mg/kg against VEGF-driven angiogenesis in KP-1/VEGF DAS assays. At 10 mg/kg, lenvatinib also inhibit FGF-driven angiogenesis in KP-1/FGF DAS assays. Sorafenib (100 mg/kg) inhibited VEGF-driven angiogenesis in the DAS assay, but did not inhibit FGF-driven in vivo angiogenesis, indicating that inhibition of VEGF signaling was not enough to inhibit FGF-driven angiogenesis in the KP-1/FGF DAS assay. FGF and VEGF synergistically [35] promote multi-step of angiogenesis. Even low levels of VEGF may promote FGF-driven angiogenesis in the KP-1/FGF DAS model. Since lenvatinib could inhibit both VEGF and FGF signaling, lenvatinib might effectively inhibited FGF-driven in vivo angiogenesis in this model. Among the FGFRs, FGFR1 and FGFR2 are likely to have the greatest impact on angiogenesis. In cell assays, sorafenib has a less potent effect on FGFR-driven proliferation (i.e. the IC_50_ value is 2 μM) [36] than does lenvatinib (the IC_50_ value is 410 nM). This less activity of sorafenib reported in cell assays might contribute to less in vivo antiangiogenesis activity in the KP-1/FGF model. VEGF-C is involved in lymphangiogenesis and lymphatic metastasis via VEGFR3 signaling [37,38] and stem cell factor (SCF) is an angiogenic factor via KIT signaling [39]. Lenvatinib inhibited SCF-induced angiogenesis and VEGF-C-induced lymphatic metastasis by inhibiting KIT and VEGFR3 [26,30]. A pan-inhibitory activity of lenvatinib against the family of multiple split kinases might contribute to lenvatinib’s broad-spectrum antitumor activity based on the inhibition of angiogenesis driven by multiple angiogenic factors such as VEGFA, SCF, VEGF-C and FGF.

Vascular score, which was the sum of MVD and pericyte coverage scores was used to analyze the association of tumor vasculature with the sensitivity of tumor models to lenvatinib treatments. There was only one model (HMV-1) with more than vascular score 8 among the 11 relatively-resistant models, but 7 out of 8 models in the sensitive group had vascular scores more than 8 (Figure 4D). This result suggested that vascular score predicted tumor shrinkage with lenvatinib treatment in preclinical 19 human tumor xenograft models. We used αSMA staining, to determine pericyte coverage in this study. But αSMA is a marker of smooth muscle cell and it may be worth to test other pericyte markers, such as NG-2, to confirm roles of pericyte in sensitivity to lenvatinib treatment in further study. Recently Smith et al. reported that IHC analysis with MVD and % of αSMA positivity identified tumor types with either tumor vessel types or stromal vessel types as phenotypes of tumor vasculature. Tumor vessel types in RCC, HCC, thyroid cancer are respond, but stromal vessel types in CRC, breast cancer, prostate cancer are refractory to a single anti-VEGF therapy [40]. Studies might be expected to confirm vascular score consisted of MVD and pericyte coverage as potential predictive biomarkers for the selection of patients responsive to antiangiogenic therapy. Since there is a large degree of heterogeneity of tumor vasculature even in each cancer type, the association of vascular score with sensitivity to antiangiogeninc therapy need to be further investigated in a larger group of tumor samples in each cancer types.

In IHC analysis of human tumor specimens, tumors in the kidney (renal cell carcinoma) had the highest MVD and the tumors in the liver (the majority was hepatocellular carcinoma (HCC)) had the lowest pericyte coverage among the 18 primary tumor types assayed. The highest vascular score was 11 for the RCC and tumors in the stomach (the majority was adenocarcinoma), followed by 10 for the lymphoma and HCC, and 9 for the thyroid (the majority was papillary thyroid cancer) and lung (non small cell lung cancer). RCC, HCC and thyroid cancer have been approved for treatment with VEGFR2 TKIs such as sunitinib (RCC), sorafenib (RCC, HCC), pazopanib (RCC), axitinib (RCC) or vandetanib (MTC). In this study, the colon and breast tumors had lower vascular scores. Bevacizumab was approved for CRC and breast cancer only in combination therapy with cytotoxic agents; and regorafenib and aflibercept [41] were also approved for CRC in combination with cytotoxic agents. Vascular scores might be useful when evaluating the indication for mono- or combo-therapy with anti-VEGF therapy.

We measured the mRNA expression levels of 59 genes related to angiogenesis in xenografted tumors with qRT-PCR by focusing on RTKs and their ligands (Additional file 1). High EGFR and low PlGF mRNA expression levels were associated with the sensitive group to lenvatinib treatments, and high expression levels of EGFR and KITLG mRNA and low expression levels of FGF7 and PDGFRA mRNA were correlated with smaller ΔT/C. The EGFR signaling pathway induced VEGF expression [42] and synergistic effects of combination therapies with EGFR inhibitors and VEGFR2 inhibitors were reported [43]. In the sTF assay, we stimulated HUVEC by using VEGF with EGF, because capillary-like-network formation was more constantly induced by the additional use of EGF. EGF may be involved in cross-talk with VEGF signaling, besides inducing VEGF expression.

## Conclusion

We showed that lenvatinib was an angiogenesis inhibitor targeting VEGFR2 and FGFR and showed antitumor activity in a broad spectrum of tumor xenograft models and vascular score consisted of high MVD and low pericyte coverage at pretreatment was the potential predictive biomarker of lenvatinib in preclinical models. The roles of tumor vasculature and vascular score as a predictive biomarker to lenvatinib treatment are warranted for further examination in clinical settings.

## Competing interests

YY, JM, TM, HO, KM, KN, OT, TS, AY, SSH, FM, TH, YF, JK, KT, MM, TW, MA, KN, TW, KY, and AT are full-time employee of Eisai Co., Ltd. ZD and YF are full-time employee of Eisai Co., Ltd. or Eisai Inc.

## Authors’ contributions

All authors contributed to the design of the study, acquisition of data, analysis and interpretation of data or manuscript writing and have read and approved the final manuscript.

## Supplementary Material

Additional file 1 **mRNA expression levels of 59 genes related to angiogenesis were associated with the antitumor activity of lenvatinib in a panel of human tumor xenograft models shown in Figure** 4**A.** We calculated ΔCT values for sample-to-sample normalization based on the mean CT values of five endogenous controls (ACTB (Hs99999903_m1), B2M (Hs99999907_m1), GAPDH (Hs99999905_m1), HMBS (Hs00609297_m1), HPRT1(Hs99999909_m1)). Fold changes were calculated after the 2-ΔCT transformation into linear space. mRNA expression levels were correlated to anti-tumor activity in xenograft model based on ΔT/C for correlation analysis and also associated with tumor response among sensitive and resistant subgroup in a panel of human tumor xenograft models divided into sensitive (ΔT/C < 0) and resistant (ΔT/C > 0) subgroups. Statistical analysis (wilcox-test and spearman correlation) was performed on the ΔCT values. Results were considered significant at p < 0.05. (A) Correlation analysis, (B) Fold difference of expression levels among relatively resistance and sensitive subgroup. Fold difference was show as median expression levels of sensitive subgroup divided by those of relatively resistant subgroup. Click here for file

Additional file 2 **Enhanced angiogenic activity of KP-1 transfectants over-expressing VEGF (human VEGF121) or FGF (mouse FGF-4) in vitro and in vivo angiogenesis assay.** (A) In vivo angiogenesis assay in mouse Dorsal Air Sac assay with KP-1 transfectants: Experiments were performed as described in materials and methods. Data are the average ± std. (B) VEGF ELISA assay: Supernatants were collected and the amounts of VEGF secreted from KP-1/VEGF determined using a VEGF ELISA Kit (Immuno-Biological Laboratories) in both normoxic (20% O2) and hypoxic (2% O2) condition. (C) Sandwich tube formation (sTF) assay using condition medium (CM) from KP-1 transfectants: sTF assay was performed using CM of KP-1 transfectants as described in materials and methods. Click here for file

Additional file 3 **Analysis of KP-1 xenpgrafts over-expressing either VEGF or FGF models in nude mice.** (A) qRT-PCR analysis of over-expression of human VEGF121 or mouse FGF4 within xenografted KP-1 transfectants. KP-1 transectants were orthotopically implanted, grown at the pancreas and then resected at the size of tumor volumes around 200 –600 mm3 (n = 4). RT-PCR; Total RNA was extracted with ISOGEN reagent (Nippongene) and cDNA was synthesized by SUPERSCRIPT first-strand synthesis systems (GIBCO BRL) with 2 mg of total DNA. PCR reaction was performed using themalcyclaer (Takara) and PCR products were electrophoresis using 2% of agarose gel and visualized with ethidium bromides. Primer information was available if requested. (B) Enhanced s.c. tumor growth of KP-1/VEGF and KP-1/FGF transfectants compared to KP-1 mock transfectants. Each group consisted of 5 mice. Data are the average ± std. dev. *p < 0.05 compared to KP-1 mock transfectants. (C) IHC analysis with H&E (upper panel) and with CD31 staining (lower panel) of endothelial cells. KP-1 transectants were orthotopically implanted and grown at the pancreas. Tumor tissues was resected 42 days after inoculation and IHC analysis was performed as described in materials and methods. Representative photographs were shown. Click here for file

Additional file 4 **Effects of lenvatinib on MVD in nude mice and on phosphorylation of VEGFR2 in KP-1/VEGF transfectant models and in HUVEC in vitro.** (A) Effects of lenvatinib on MVD within KP-1/VEGF xenografted tumors. Lenvatinib was administered orally twice daily at indicated doses. Tumor tissues were resected after 14 days treatment. IHC analysis of MVD was performed with anti-mouse CD31 antibody, as described in materials and methods. Each group consisted of 5 mice. Data are the average ± std. **p < 0.01 compared to vehicle. (B-C) Western blotting (WB) analysis for phosphorylated proteins. (B) Effects of lenvatinib on phosphorylation of VEGFR2 within KP-1/VEGF xenografted tumors. Lenvatinib was administered at either 3 or 30 mg/kg in mice (n = 3) bearing KP-1/VEGF xenografted tumors. Tumors were resected at indicated times after lenvatinib administrations. Tumor was crushed by homogenizer with lysis buffer including phosphatase inhibitor and then prepared adequate concentration of lysate protein was subjected to SDS-PAGE. KP-1/VEGF cells do not express VEGFR2 (data not shown). (C) Western blotting analysis of VEGF-stimulated phosphorylation of VEGFR2 and downstream molecules in HUVECs. HUVECs were grown to subconfluence and then starved with human endothelial serum-free medium (SFM) basal medium containing 0.5% FBS for 24 hrs. HUVECs were treated with the indicated concentrations of lenvatinib for 60 min, followed by VEGF stimulation (20 ng/mL) for 5 min. Primary antibodies against VEGFR2, phospho-VEGFR2, Erk1/2, phospho-Erk1/2, Akt and phospho-Akt (Cell Signaling Technology; 1:1000) and the secondary antibody, anti-Rabbit IgG (H&L) HRP-linked antibody (Cell Signaling Technology; 1:1000) were used. The blots were developed with SuperSignal West Pico chemiluminescent substrate (Pierce). Immunoreactive bands were visualized by chemiluminescence with an Image Master™ VDS-CL detection system (Amersham Pharmacia Biotech). Click here for file

Additional file 5 **Summary of antitumor activity of lenvatinib and tumor vasculature in a panel of human tumor xenograft models in nude mice. Lenvatinib was administered orally twice daily for 7 days at 100 mg/kg.** Each group consisted of 3–6 mice. IHC analysis of microvessel density (MVD) and % of pericyte coverage were performed by staining CD31 and aSMA. ΔT/C (%) was shown as a mean and both MVD and pericyte coverage were shown as a median. Scoring of microvessel density (MVD) and pericyte coverage was based on a table as shown in Additional file 6A. Vascular scores were the sum of MVD and pericyte coverage scores. Antiangiogenesis activity was show as % of means of MVD and pericyte coverage at post-treatment (day 8) compared to those at pretreatment. Click here for file

Additional file 6 **Scoring for microvessel density (MVD) and % of pericyte coverage by IHC analysis of with staining CD31 and aSMA based on the median values of each group analysis.** Analysis was performed as descried in materials and methods. (A) Scores for 19 human tumor xenograft models, (B) Scored for 18 types of tumor tissues specimens. Click here for file

Additional file 7 **Summary of human tumor specimens among 18 different types.** ad-ca; adenocarcinoma, rcc; renal cell carcinoma, scc; squamous cell carcinoma. Click here for file

Additional file 8 **IHC Analysis for Microvessel density (MVD) and % of pericyte covered vessels in human tumor specimens with MVD and pericyte coverage scores.** MVD and pericyte coverage scores (Additional file 6B) were based on the median values of analysis from each type of tumor tissue specimens. Click here for file
