# Supplementary material for: Lenvatinib, an angiogenesis inhibitor targeting VEGFR/FGFR, shows broad antitumor activity in human tumor xenograft models associated with microvessel density and pericyte coverage
Source: Vasc Cell. 2014 Sep 6;6:18. doi: 10.1186/2045-824X-6-18 (PMC4156793; doi:10.1186/2045-824X-6-18)
Supplement: Additional file 1 — mRNA expression levels of 59 genes related to angiogenesis were associated with the antitumor activity of lenvatinib in a panel of human tumor xenograft models shown in Figure 4A. We calculated ΔCT values for sample-to-sample normalization based on the mean CT values of five endogenous controls (ACTB (Hs99999903_m1), B2M (Hs99999907_m1), GAPDH (Hs99999905_m1), HMBS (Hs00609297_m1), HPRT1(Hs99999909_m1)). Fold changes were calculated after the 2-ΔCT transformation into linear space. mRNA expression levels were correlated to anti-tumor activity in xenograft model based on ΔT/C for correlation analysis and also associated with tumor response among sensitive and resistant subgroup in a panel of human tumor xenograft models divided into sensitive (ΔT/C < 0) and resistant (ΔT/C > 0) subgroups. Statistical analysis (wilcox-test and spearman correlation) was performed on the ΔCT values. Results were considered significant at p < 0.05. (A) Correlation analysis, (B) Fold difference of expression levels among relatively resistance and sensitive subgroup. Fold difference was show as median expression levels of sensitive subgroup divided by those of relatively resistant subgroup. [file 2045-824X-6-18-S1.pdf]

Additional file 1

A. Correlation analysis

Gene	Probe	Correlation	
		R	p value
PDGFRA	Hs00183486_m1	-0.590	0.010
EGFR	Hs00193306_m1	0.568	0.014
KITLG	Hs00241497_m1	0.533	0.023
FGF 7	Hs00384281_m1	-0.470	0.049
PIGF	Hs00601696_m1	-0.459	0.055
PDGFRB	Hs00182163_m1	-0.427	0.077
ANGPT1	Hs00181613_m1	-0.422	0.081
HGF	Hs00300159_m1	-0.419	0.084
FGF21	Hs00173927_m1	0.403	0.097
FLT4	Hs01047687_g1	0.398	0.102
TEK	Hs00945146_m1	-0.388	0.112
RET	Hs01120030_m1	0.386	0.114
VEGFA	Hs00900055_m1	0.367	0.134
PTN	Hs01085691_m1	-0.360	0.142
FGFR2	Hs00256527_m1	0.348	0.157
IL6	Hs00985641_m1	0.347	0.158
TNF	Hs00174128_m1	0.337	0.171
FGFR1	Hs00915134_g1	0.323	0.191
FGFR3	Hs01005393_m1	0.322	0.193
FGFR3	Hs00179829_m1	0.304	0.220
MET	Hs00179845_m1	0.273	0.273
FGFR1	Hs00917384_m1	0.256	0.306
IGF2	Hs00171254_m1	-0.253	0.312
VEGFC	Hs01099203_m1	-0.217	0.386
FGF18	Hs00818572_m1	0.215	0.392
FGFR1	Hs00241111_m1	0.211	0.400
FGFR1	Hs00915137_m1	0.188	0.454
FIGF	Hs01128659_m1	-0.186	0.461
IL8	Hs00174103_m1	-0.184	0.464
PDGFB	Hs00966522_m1	0.184	0.466
FGF19	Hs00192780_m1	0.167	0.508
FGF16	Hs00175752_m1	-0.164	0.515
VEGFB	Hs00173634_m1	0.151	0.550
FGF 9	Hs00181829_m1	-0.149	0.556
FGFR3	Hs00997397_m1	0.129	0.610
FGF 8	Hs00171832_m1	0.123	0.626
FGFR2	Hs00240792_m1	0.123	0.628
FLT1	Hs01904119_gH	0.121	0.633
ANGPT2	Hs01048042_m1	0.115	0.648
IGF1	Hs01547656_m1	0.113	0.655
PDGFA	Hs00964426_m1	0.110	0.665
FGFR4	Hs01107438_m1	0.109	0.668
FGF 5	Hs03676587_s1	-0.106	0.677
GDNF	Hs01931883_s1	-0.106	0.677
TGFB1	Hs00998133_m1	0.101	0.689
FGF 1	Hs00265254_m1	-0.100	0.694
FGF20	Hs00173929_m1	-0.099	0.696
HBEGF	Hs00181813_m1	0.098	0.698
KIT	Hs00174029_m1	-0.096	0.706
FGF22	Hs00221001_m1	0.092	0.716
FGF17	Hs00182599_m1	0.090	0.723
FGF 2	Hs00266645_m1	-0.078	0.759
FGF 3	Hs00173742_m1	0.075	0.768
KDR	Hs00176676_m1	-0.045	0.858
FGFR4	Hs00608745_m1	0.040	0.873
FGFR4	Hs00242558_m1	-0.005	0.984
FGF 4	Hs00173564_m1	NA	NA
FGF 6	Hs00173934_m1	NA	NA
FGF10	Hs00610298_m1	NA	NA

B. Fold difference of expression levels among resistance and sensitive subgroups

Gene	Probe	Correlation		Median expression (2^{-dCT})	
		Relatively resistant	Sensitive	Fold difference	p value
EGFR	Hs00193306_m1	0.025	0.173	6.867	0.018
PIGF	Hs00601696_m1	0.012	0.007	0.554	0.027
TNF	Hs00174128_m1	0.001	0.001	1.194	0.074
KITLG	Hs00241497_m1	0.012	0.021	1.757	0.085
PDGFRA	Hs00183486_m1	0.001	NA	NA	0.093
IL6	Hs00985641_m1	0.007	0.003	0.391	0.122
FGF 7	Hs00384281_m1	0.000	0.001	1.817	0.169
VEGFA	Hs00900055_m1	0.045	0.081	1.812	0.179
HBEGF	Hs00181813_m1	0.019	0.043	2.270	0.246
FLT4	Hs01047687_g1	NA	0.003	NA	0.254
IGF2	Hs00171254_m1	0.005	0.001	0.250	0.263
HGF	Hs00300159_m1	0.017	NA	NA	0.281
FGFR2	Hs00256527_m1	0.006	0.001	0.203	0.287
RET	Hs01120030_m1	0.002	0.000	0.240	0.320
TGFB1	Hs00998133_m1	0.383	0.698	1.822	0.328
IGF1	Hs01547656_m1	0.000	0.000	0.940	0.329
PDGFRB	Hs00182163_m1	0.001	0.001	1.241	0.345
FGFR4	Hs01107438_m1	0.002	0.005	2.212	0.375
FGF 9	Hs00181829_m1	0.003	0.004	1.412	0.398
FLT1	Hs01904119_gH	0.004	0.002	0.435	0.413
TEK	Hs00945146_m1	0.000	0.000	1.691	0.448
FGFR3	Hs00179829_m1	0.003	0.002	0.699	0.468
FGFR4	Hs00608745_m1	0.002	0.005	2.787	0.468
ANGPT1	Hs00181613_m1	0.015	0.004	0.299	0.477
FGF17	Hs00182599_m1	0.001	0.000	0.449	0.483
FGF21	Hs00173927_m1	0.001	0.001	0.910	0.489
PTN	Hs01085691_m1	0.025	0.003	0.127	0.489
FGF16	Hs00175752_m1	0.000	NA	NA	0.494
FGF18	Hs00818572_m1	0.000	0.000	1.770	0.525
MET	Hs00179845_m1	0.089	0.084	0.944	0.536
FGF 1	Hs00265254_m1	0.001	0.002	2.034	0.586
FGFR1	Hs00915134_g1	0.025	0.025	1.007	0.596
PDGFA	Hs00964426_m1	0.025	0.020	0.813	0.596
FGF19	Hs00192780_m1	0.004	0.001	0.209	0.636
FGFR3	Hs00997397_m1	0.001	0.005	3.779	0.682
FGF 2	Hs00266645_m1	0.012	0.009	0.758	0.683
FGF20	Hs00173929_m1	0.000	0.000	0.893	0.691
FGFR3	Hs01005393_m1	0.002	0.002	1.164	0.699
FGFR1	Hs00917384_m1	0.023	0.018	0.768	0.724
FGFR4	Hs00242558_m1	0.017	0.019	1.156	0.724
IL8	Hs00174103_m1	0.036	0.021	0.592	0.724
VEGFB	Hs00173634_m1	0.068	0.100	1.481	0.724
FGF 8	Hs00171832_m1	0.000	0.001	1.568	0.785
VEGFC	Hs01099203_m1	0.006	0.008	1.434	0.852
FIGF	Hs01128659_m1	0.002	0.001	0.623	0.856
FGF 3	Hs00173742_m1	0.181	0.000	0.001	0.868
KIT	Hs00174029_m1	0.179	0.000	0.002	0.914
FGFR1	Hs00915137_m1	0.010	0.008	0.820	0.928
PDGFB	Hs00966522_m1	0.014	0.007	0.456	0.928
FGF 5	Hs03676587_s1	0.001	0.002	3.114	0.964
ANGPT2	Hs01048042_m1	0.001	0.002	1.212	1.000
FGF22	Hs00221001_m1	0.000	0.000	0.937	1.000
FGFR1	Hs00241111_m1	0.033	0.035	1.053	1.000
FGFR2	Hs00240792_m1	0.001	0.006	4.062	1.000
GDNF	Hs01931883_s1	0.003	0.002	0.565	1.000
KDR	Hs00176676_m1	0.001	0.004	3.373	1.000
FGF 4	Hs00173564_m1	NA	NA	NA	NA
FGF 6	Hs00173934_m1	NA	NA	NA	NA
FGF10	Hs00610298_m1	NA	NA	NA	NA
