# Supplementary figures and images for: Lenvatinib, an angiogenesis inhibitor targeting VEGFR/FGFR, shows broad antitumor activity in human tumor xenograft models associated with microvessel density and pericyte coverage
Source: Vasc Cell. 2014 Sep 6;6:18. doi: 10.1186/2045-824X-6-18 (PMC4156793; doi:10.1186/2045-824X-6-18)

**A**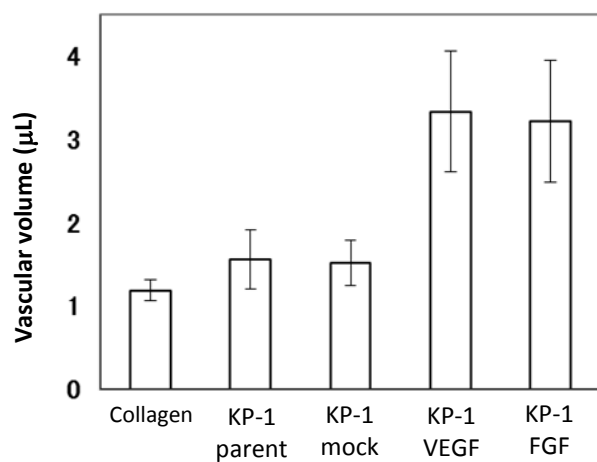**B**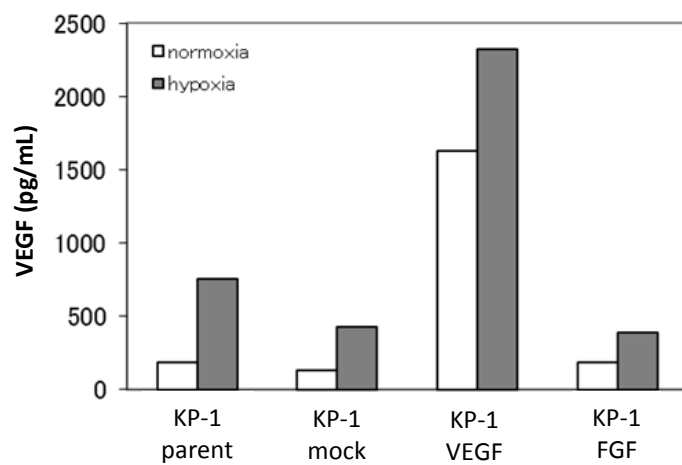**C**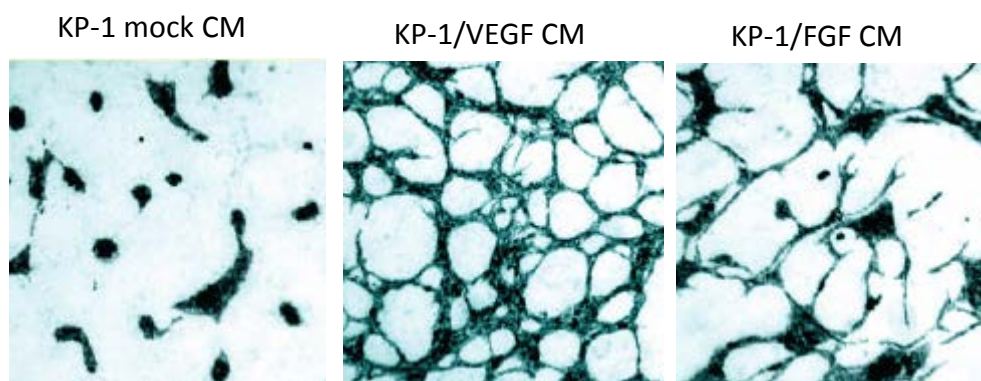

Supplement: Additional file 2 — Enhanced angiogenic activity of KP-1 transfectants over-expressing VEGF (human VEGF121) or FGF (mouse FGF-4) in vitro and in vivo angiogenesis assay. (A) In vivo angiogenesis assay in mouse Dorsal Air Sac assay with KP-1 transfectants: Experiments were performed as described in materials and methods. Data are the average ± std. (B) VEGF ELISA assay: Supernatants were collected and the amounts of VEGF secreted from KP-1/VEGF determined using a VEGF ELISA Kit (Immuno-Biological Laboratories) in both normoxic (20% O2) and hypoxic (2% O2) condition. (C) Sandwich tube formation (sTF) assay using condition medium (CM) from KP-1 transfectants: sTF assay was performed using CM of KP-1 transfectants as described in materials and methods. [file 2045-824X-6-18-S2.pdf]

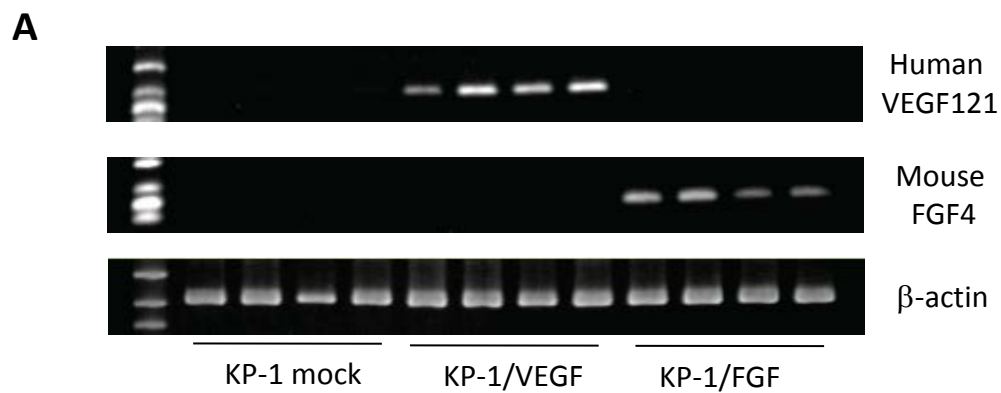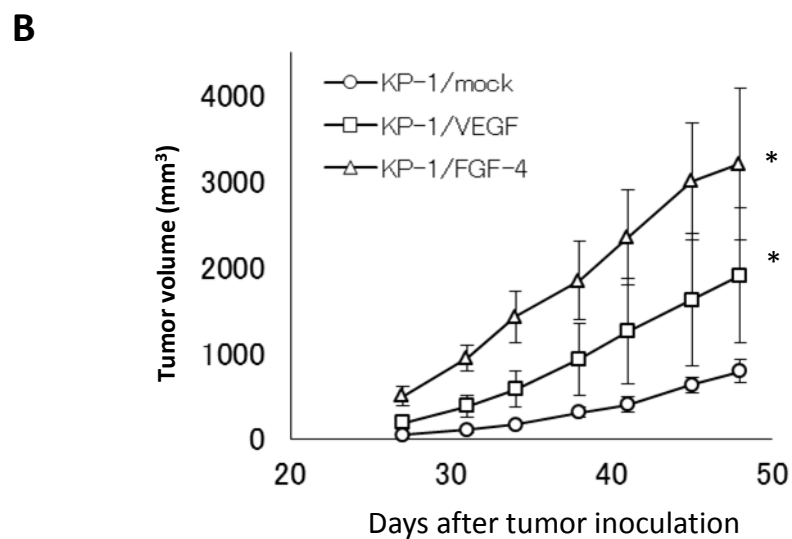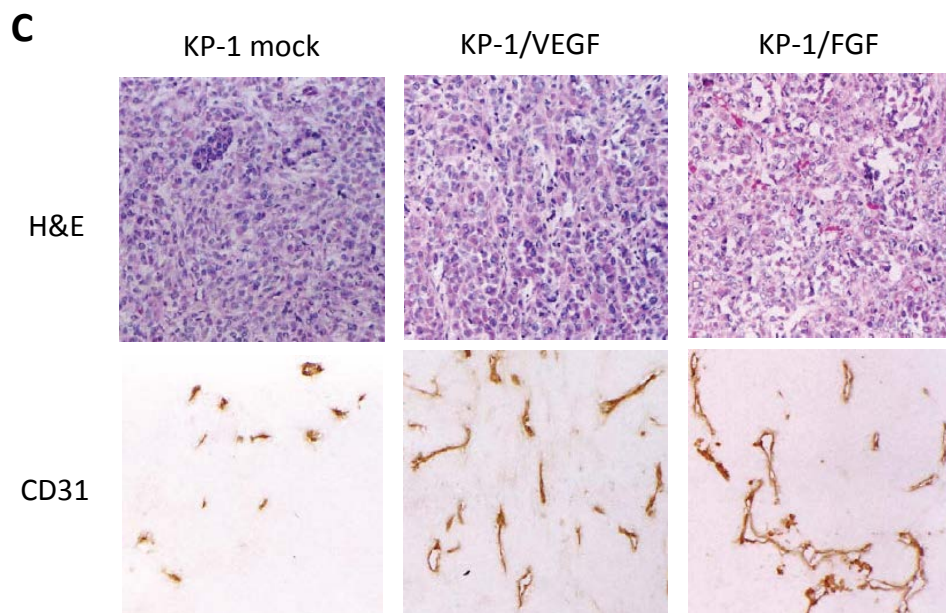

Supplement: Additional file 3 — Analysis of KP-1 xenpgrafts over-expressing either VEGF or FGF models in nude mice. (A) qRT-PCR analysis of over-expression of human VEGF121 or mouse FGF4 within xenografted KP-1 transfectants. KP-1 transectants were orthotopically implanted, grown at the pancreas and then resected at the size of tumor volumes around 200 –600 mm3 (n = 4). RT-PCR; Total RNA was extracted with ISOGEN reagent (Nippongene) and cDNA was synthesized by SUPERSCRIPT first-strand synthesis systems (GIBCO BRL) with 2 mg of total DNA. PCR reaction was performed using themalcyclaer (Takara) and PCR products were electrophoresis using 2% of agarose gel and visualized with ethidium bromides. Primer information was available if requested. (B) Enhanced s.c. tumor growth of KP-1/VEGF and KP-1/FGF transfectants compared to KP-1 mock transfectants. Each group consisted of 5 mice. Data are the average ± std. dev. *p < 0.05 compared to KP-1 mock transfectants. (C) IHC analysis with H&E (upper panel) and with CD31 staining (lower panel) of endothelial cells. KP-1 transectants were orthotopically implanted and grown at the pancreas. Tumor tissues was resected 42 days after inoculation and IHC analysis was performed as described in materials and methods. Representative photographs were shown. [file 2045-824X-6-18-S3.pdf]

**A**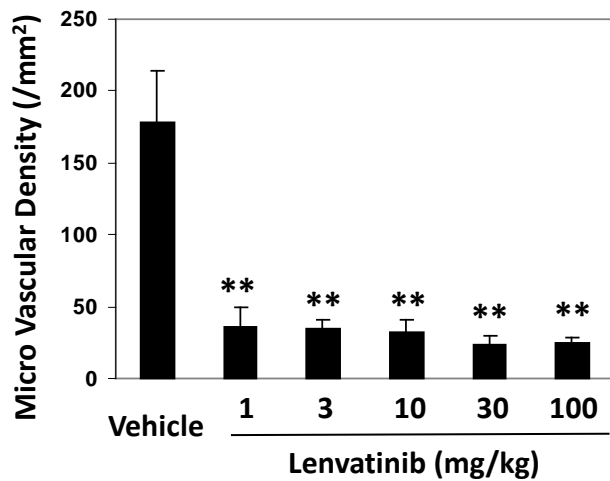**B**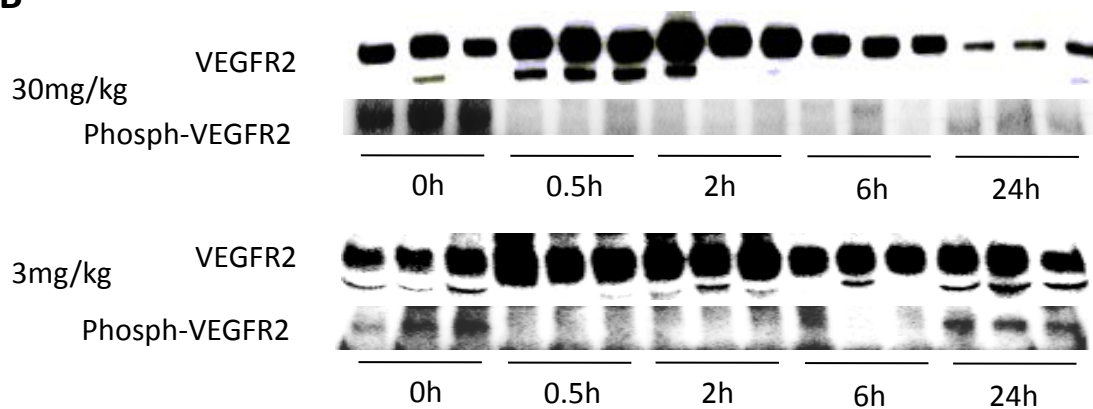**C**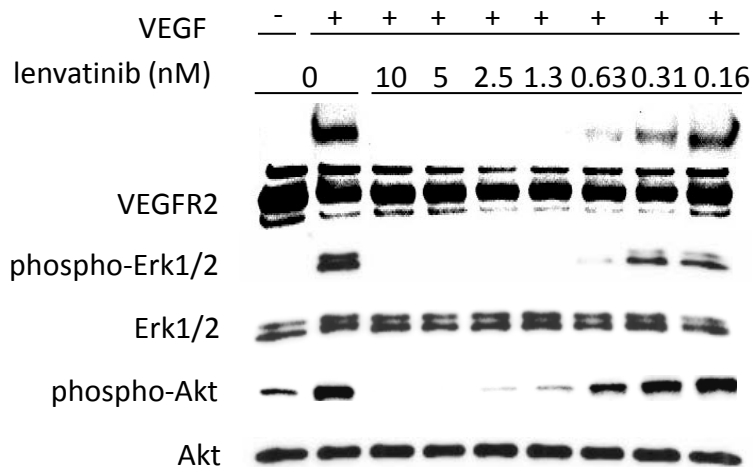

Supplement: Additional file 4 — Effects of lenvatinib on MVD in nude mice and on phosphorylation of VEGFR2 in KP-1/VEGF transfectant models and in HUVEC in vitro. (A) Effects of lenvatinib on MVD within KP-1/VEGF xenografted tumors. Lenvatinib was administered orally twice daily at indicated doses. Tumor tissues were resected after 14 days treatment. IHC analysis of MVD was performed with anti-mouse CD31 antibody, as described in materials and methods. Each group consisted of 5 mice. Data are the average ± std. **p < 0.01 compared to vehicle. (B-C) Western blotting (WB) analysis for phosphorylated proteins. (B) Effects of lenvatinib on phosphorylation of VEGFR2 within KP-1/VEGF xenografted tumors. Lenvatinib was administered at either 3 or 30 mg/kg in mice (n = 3) bearing KP-1/VEGF xenografted tumors. Tumors were resected at indicated times after lenvatinib administrations. Tumor was crushed by homogenizer with lysis buffer including phosphatase inhibitor and then prepared adequate concentration of lysate protein was subjected to SDS-PAGE. KP-1/VEGF cells do not express VEGFR2 (data not shown). (C) Western blotting analysis of VEGF-stimulated phosphorylation of VEGFR2 and downstream molecules in HUVECs. HUVECs were grown to subconfluence and then starved with human endothelial serum-free medium (SFM) basal medium containing 0.5% FBS for 24 hrs. HUVECs were treated with the indicated concentrations of lenvatinib for 60 min, followed by VEGF stimulation (20 ng/mL) for 5 min. Primary antibodies against VEGFR2, phospho-VEGFR2, Erk1/2, phospho-Erk1/2, Akt and phospho-Akt (Cell Signaling Technology; 1:1000) and the secondary antibody, anti-Rabbit IgG (H&L) HRP-linked antibody (Cell Signaling Technology; 1:1000) were used. The blots were developed with SuperSignal West Pico chemiluminescent substrate (Pierce). Immunoreactive bands were visualized by chemiluminescence with an Image Master™ VDS-CL detection system (Amersham Pharmacia Biotech). [file 2045-824X-6-18-S4.pdf]
