# Supplementary material for: Lenvatinib, an angiogenesis inhibitor targeting VEGFR/FGFR, shows broad antitumor activity in human tumor xenograft models associated with microvessel density and pericyte coverage
Source: Vasc Cell. 2014 Sep 6;6:18. doi: 10.1186/2045-824X-6-18 (PMC4156793; doi:10.1186/2045-824X-6-18)
Supplement: Additional file 5 — Summary of antitumor activity of lenvatinib and tumor vasculature in a panel of human tumor xenograft models in nude mice. Lenvatinib was administered orally twice daily for 7 days at 100 mg/kg. Each group consisted of 3–6 mice. IHC analysis of microvessel density (MVD) and % of pericyte coverage were performed by staining CD31 and aSMA. ΔT/C (%) was shown as a mean and both MVD and pericyte coverage were shown as a median. Scoring of microvessel density (MVD) and pericyte coverage was based on a table as shown in Additional file 6A. Vascular scores were the sum of MVD and pericyte coverage scores. Antiangiogenesis activity was show as % of means of MVD and pericyte coverage at post-treatment (day 8) compared to those at pretreatment. [file 2045-824X-6-18-S5.pdf]

Additional file 5

Cell line	Tumor type	Antitumor activity		Tumor vascularity at pre-treatment					Antiangiogenesis activity (T/C, %)	
		ΔT/C (%)	Subgroup	MVD		Pericyte coverage		Vascular score	MVD	Pericyte coverage
				/mm2	Score	%	Score			
DU145	Prostate	-60%	sensitive	93.0	4	11.0	4	8	22.0	1300.0
AZ-521	Stomach	-57%		158.4	6	14.6	4	10	123.5	154.1
MDA-468	Breast	-53%		80.0	4	22.7	2	6	41.9	132.0
MDA-231	Breast	-33%		149.0	6	6.0	5	10	20.0	400.0
Lovo	Colon	-11%		95.0	4	11.0	4	8	42.0	175.0
AsPC-1	Pancreas	-8%		108.0	5	8.0	5	10	39.0	300.0
A431	Skin	-8%		55.0	3	10.0	5	8	26.0	241.0
SK-Mel-2	Melanoma	-3%		79.9	4	10.0	5	9	50.7	326.0
HCT116	Colon	17%	relatively resistant	50.5	3	31.6	0	3	60.6	111.0
HMV-1	Melanoma	19%		131.5	6	24.7	2	8	52.5	69.0
MDA-MB-435	Melanoma	22%		55.5	3	37.7	0	3	85.9	177.0
SW620	Colon	22%		44.3	2	20.6	2	4	63.9	154.0
H526	SCLC	22%		55.0	3	81.0	0	3	50.0	121.0
A375	Melanoma	26%		90.9	4	20.9	2	6	54.6	174.0
LOX	Melanoma	42%		88.2	4	30.0	1	5	33.2	219.0
DLD-1	Colon	52%		76.6	4	29.5	1	5	43.8	125.0
PC-3	Prostate	54%		51.0	3	16.0	3	6	48.4	238.0
FEM	Melanoma	55%		39.3	2	36.0	0	2	95.9	174.0
SEKI	Melanoma	110%		92.8	4	18.1	3	7	36.4	132.4
