# Supplementary material for: Lenvatinib, an angiogenesis inhibitor targeting VEGFR/FGFR, shows broad antitumor activity in human tumor xenograft models associated with microvessel density and pericyte coverage
Source: Vasc Cell. 2014 Sep 6;6:18. doi: 10.1186/2045-824X-6-18 (PMC4156793; doi:10.1186/2045-824X-6-18)
Supplement: Additional file 6 — Scoring for microvessel density (MVD) and % of pericyte coverage by IHC analysis of with staining CD31 and aSMA based on the median values of each group analysis. Analysis was performed as descried in materials and methods. (A) Scores for 19 human tumor xenograft models, (B) Scored for 18 types of tumor tissues specimens. [file 2045-824X-6-18-S6.pdf]

Additional file 6

A. Xenograft tissue

Score	MVD (per mm^2)		Pericyte coverage (%)	
	over	less than (≤)	over	less than (≤)
6	125	-	0	5
5	100	125	5	10
4	75	100	10	15
3	50	75	15	20
2	25	50	20	25
1	0	25	25	30
0	-	-	30	-

B. Human tumor specimens

Score	MVD (per mm^2)		Pericyte coverage (%)	
	over	less than (≤)	over	less than (≤)
6	500	600	0	5
5	400	500	5	10
4	300	400	10	15
3	200	300	15	20
2	100	200	20	25
1	0	100	25	30
0	-	-	30	-
