# Supplementary material for: Lenvatinib, an angiogenesis inhibitor targeting VEGFR/FGFR, shows broad antitumor activity in human tumor xenograft models associated with microvessel density and pericyte coverage
Source: Vasc Cell. 2014 Sep 6;6:18. doi: 10.1186/2045-824X-6-18 (PMC4156793; doi:10.1186/2045-824X-6-18)
Supplement: Additional file 7 — Summary of human tumor specimens among 18 different types. ad-ca; adenocarcinoma, rcc; renal cell carcinoma, scc; squamous cell carcinoma. [file 2045-824X-6-18-S7.pdf]

Additional file 7

Tumor in the organ	N	Diagnosis	N
Stomach	10	poorly differentiated ad-ca	4
		mucinous adenocarcinoma	2
		moderately differentiated ad-ca	2
		Signet ring cell	1
		Mucinous ad-ca & signet ring cell	1
Esophagus	10	moderately differentiated scc	8
		well differentiated scc	2
Lung	10	bronchogenic scc	7
		well differentiated ad-ca	2
		large cell ca	1
Colon	10	rectum	6
		transverse colon	2
		sigmoid colon	1
		cecum	1
Thyroid	10	papillary	9
		anaplastic	1
Kidney	9	clear cell rcc	5
		undifferentiated rcc	1
		granular cell rcc	1
		oncotic variant rcc	1
		rcc	1
Breast	10	infiltrating duct carcinoma	6
		infiltrating papillary carcinoma with signet ring cell	1
		mixed infiltrating duct and lobular carcinoma	1
		medullary	1
		atypical medullary	1
Neck, Bone, Jejunum, cecum, stomach, retroperitoneum, testis, Duodenum	10	malignant lymphoma	-
Liver	10	hepatocellular carcinoma	8
		combined hepatocellular and cholangiocarcinoma	2
Urinary bladder	10	transitional cell carcinoma	6
		papillary transitional cell carcinoma	3
		mucinous adenocarcinoma from urachal remnant	1
Ovary	10	papillary serous cystadeno carcinoma	9
		mucinous cystadeno carcinoma	1
Pancreas	10	moderately differentiated ductal carcinoma	9
		poorly differentiated ductal carcinoma	1
Prostate	9	adenocarcinoma	9
Endometirum	10	adenocarcinoma endometroid type	5
		adenocarcinoma	4
		adenocarcinoma extension from uterine cervix	1
Gallbladder	10	well differentiated ad-ca	4
		moderately differentiated ad-ca	3
		poorly differentiated ad-ca	1
		metastatic ad-ca from stomach	1
		adenosquamous carcinoma	1
Head and neck (larynx, epiglottis, oral cavity)	10	well differentiated scc	5
		moderately differentiated scc	2
		poorly differentiated scc	1
		adenoid cyctic carcinoma	1
		scc	1
Utrerine cervix	10	scc	9
		adenosquamous carcinoma	1
Skin (sole, toe, scalp, hip, abdemen, buttock, axilla, face), anus	9	malignant melanoma	-
