# Supplementary material for: Lenvatinib, an angiogenesis inhibitor targeting VEGFR/FGFR, shows broad antitumor activity in human tumor xenograft models associated with microvessel density and pericyte coverage
Source: Vasc Cell. 2014 Sep 6;6:18. doi: 10.1186/2045-824X-6-18 (PMC4156793; doi:10.1186/2045-824X-6-18)
Supplement: Additional file 8 — IHC Analysis for Microvessel density (MVD) and % of pericyte covered vessels in human tumor specimens with MVD and pericyte coverage scores. MVD and pericyte coverage scores (Additional file 6B) were based on the median values of analysis from each type of tumor tissue specimens. [file 2045-824X-6-18-S8.pdf]

Additional file 8

A. Microvessel density (per mm^2)

Tumor in the organ	Median	Range	Score
Kidney	595.5	266 - 1500	6
Stomach	561.5	118 - 1082	
Endometrium	443.5	55 - 1222	5
Thyroid	438.5	135 - 884	
Lymphoma	434.0	195 - 756	
Gallbladder	411.0	12 - 869	
Lung	407.5	169 - 693	
Head and neck	334.5	162 - 666	4
Uterine cervical	321.5	110 - 815	
Liver	307.0	15 - 1500	
Breast	294.0	144 - 713	3
Pancreas	288.0	149 - 548	
Urinary bladder	284.5	179 - 372	
Prostate	274.5	226 - 745	
Colon	250.0	164 - 830	
Melanoma	221.0	116 - 488	
Esophagus	204.0	90 - 601	
Ovary	185.5	75 - 599	2

B. Pericyte coverage (%)

Tumor in the organ	Median	Range	Score
Liver	4.9	0.0 - 38.4	6
Lymphoma	5.7	3.0 - 20.5	5
Kidney	7.4	1.5 - 78.6	
Stomach	7.6	2.0 - 30.2	
Esophagus	7.8	0.0 - 9.6	
Ovary	9.6	0.0 - 27.8	
Pancreas	10	3.0 - 23.7	4
Thyroid	10.6	2.6 - 30.4	
Head and neck	10.9	2.4 - 40.7	
Lung	11.1	1.1 - 20.7	
Urinary bladder	11.8	1.4 - 20.8	
Uterine cervix	11.9	3.0 - 34.9	
Endometrium	16.1	2.4 - 78.2	3
Gallbladder	22.1	6.7 - 49.0	2
Colon	24.9	8.3 - 43.4	
Prostate	26.4	10.2 - 79.5	1
Breast	28	9.7 - 54.7	
Melanoma	29.6	0.6 - 51.6	
